# Metformin Affects Cardiac Arachidonic Acid Metabolism and Cardiac Lipid Metabolite Storage in a Prediabetic Rat Model

**DOI:** 10.3390/ijms22147680

**Published:** 2021-07-19

**Authors:** Denisa Miklankova, Irena Markova, Martina Hüttl, Iveta Zapletalova, Martin Poruba, Hana Malinska

**Affiliations:** 1 Centre for Experimental Medicine, Institute for Clinical and Experimental Medicine, 14021 Prague, Czech Republic; denisa.miklankova@ikem.cz (D.M.); irena.markova@ikem.cz (I.M.); martina.huttl@ikem.cz (M.H.); 2 First Faculty of Medicine, Charles University, 12108 Prague, Czech Republic; 3 Department of Pharmacology, Faculty of Medicine and Dentistry, Palacky University Olomouc, 77900 Olomouc, Czech Republic; iveta.zapletalova@upol.cz (I.Z.); martin.poruba@upol.cz (M.P.)

**Keywords:** metformin, stearoyl-CoA desaturase, fatty acid profile, myocardial phospholipids, arachidonic acid, cytochrome P450, myocardial function, lipotoxic intermediates

## Abstract

Metformin can reduce cardiovascular risk independent of glycemic control. The mechanisms behind its non-glycemic benefits, which include decreased energy intake, lower blood pressure and improved lipid and fatty acid metabolism, are not fully understood. In our study, metformin treatment reduced myocardial accumulation of neutral lipids—triglycerides, cholesteryl esters and the lipotoxic intermediates—diacylglycerols and lysophosphatidylcholines in a prediabetic rat model (*p* < 0.001). We observed an association between decreased gene expression and SCD-1 activity (*p* < 0.05). In addition, metformin markedly improved phospholipid fatty acid composition in the myocardium, represented by decreased SFA profiles and increased n3-PUFA profiles. Known for its cardioprotective and anti-inflammatory properties, metformin also had positive effects on arachidonic acid metabolism and CYP-derived arachidonic acid metabolites. We also found an association between increased gene expression of the cardiac isoform CYP2c with increased 14,15-EET (*p* < 0.05) and markedly reduced 20-HETE (*p* < 0.001) in the myocardium. Based on these results, we conclude that metformin treatment reduces the lipogenic enzyme SCD-1 and the accumulation of the lipotoxic intermediates diacylglycerols and lysophosphatidylcholine. Increased CYP2c gene expression and beneficial effects on CYP-derived arachidonic acid metabolites in the myocardium can also be involved in cardioprotective effect of metformin.

## 1. Introduction

Metabolic syndrome (MS) and prediabetes are characterized by a group of disturbances associated with visceral obesity, impaired glucose signaling pathways, altered lipid metabolism, insulin resistance (IR), hypertension and hepatic steatosis [1]. The pathogenesis of these metabolic disorders is influenced by alterations in the metabolism, and the synthesis and regulation of fatty acids (FA). These changes in FA metabolism are accompanied by IR and adipose tissue dysregulation, both of which are independent cardiovascular risk factors of MS and prediabetes development [2].

The hallmarks of FA alterations in MS and prediabetes are decreased long-chain polyunsaturated fatty acid (PUFA) profiles and disorders of desaturation and elongation enzymes [3]. This enzyme system is involved in the regulation of whole-body metabolism, glucose and lipid metabolism, and insulin sensitivity [4]. MS is typically characterized by decreased linoleic acid (LA, 18:2n6) and n3-PUFA profiles in phospholipids (PL) and increased stearoyl-CoA desaturase (SCD), a key lipogenic enzyme. SCD regulates myocardial metabolism and influences substrate utilization, which can in turn affect cardiac function [5,6]. Recent studies have investigated both the beneficial and detrimental effects of bioactive lipid metabolites generated by PUFA, in particular arachidonic acid (AA, 20:4n6), in order to elucidate their role in cardiac function and disease [7]. Other research efforts have helped to reveal the important effects of cytochrome (CYP) P450-derived epoxygenase and hydroxylase metabolites of AA in relation to cardiac function and metabolism. Thus, alterations and disorders in FA metabolism and regulation are considered promising therapeutic targets in the treatment of MS and cardiovascular complications.

Metformin is a first-line antidiabetic drug used to mitigate cardiovascular risk and total mortality in the treatment of type 2 diabetes. It is well known that metformin improves glycemic control by suppressing hepatic gluconeogenesis and has beneficial effects on insulin sensitivity, body weight, blood pressure and lipid metabolism [8]. According to the UK Prospective Diabetes Study, and more recent retrospective studies, metformin treatment reduces the risk of myocardial infarction, stroke, atrial fibrillation and all-cause mortality [9]. The results of these studies indicate that metformin-induced improvements in cardiovascular outcomes are independent of glycemic control. Exhibiting numerous non-glycemic effects, metformin is also indicated for use in a number of novel drug targets. The mechanisms behind its non-glycemic benefits, which include decreased energy intake, lower blood pressure and improved lipid and fatty acid metabolism, are not fully understood [9,10]. Metformin can improve lipid metabolism by reducing hepatic steatosis, as demonstrated in one rodent model [11] and another clinical study [12]. According to recent reports, metformin has a beneficial effect on circulating lipids by lowering plasma triglycerides (TG) [13], with the drug also attenuating hepatic lipid accumulation in patients with non-alcoholic fatty liver disease (NAFLD) [14].

Experimental studies have shown that metformin favorably affects cardiac energetic metabolism, structure and function by decreasing inflammation as well as oxidative and dicarbonyl stress and improving endothelial function [15,16]. Metformin through activation of AMP-kinase shows cardioprotective effects during cardiac remodeling, hypertrophy and fibrosis. New studies consider the cardioprotective and therapeutic potential of metformin in focusing beyond AMP-kinase, specially targeting autophagy, mitochondrial function and mRNA [17]. In addition, metformin is understood to be a possible inhibitor of SCD, significantly affecting FA composition in circulating non-esterified fatty acids (NEFA), as well as metabolic active tissues [15,18]. However, precisely how metformin affects FA metabolism and regulation in the myocardium is unclear.

In this study, we investigated the protective effects of metformin on cardiac lipid and FA metabolism and dysregulation in a prediabetic rat model: hereditary hypertriglyceridemic (HHTg) rats. This particular rat strain is characterized by the presence of severe genetically determined hypertriglyceridemia, IR and hepatic steatosis and the absence of obesity and fasting hyperglycemia [19].

## 2. Results

### 2.1. Characterization of Cardiac Lipid Metabolic Parameters in HHTg Rats

Compared to Wistar controls, untreated HHTg rats exhibited markedly increased serum TG, cholesterol, and NEFA, and decreased HDL-C (Table 1 and Table S1). While body weight was not affected in HHTg rats, relative weight of epididymal adipose tissue (EAT) significantly increased compared to Wistar controls. In addition, hypertriglyceridemia in HHTg rats was associated with impaired glucose tolerance—non-fasting glucose, AUC_0-180min_ and HOMA-IR were markedly elevated. We observed a significant decrease of glucagon in untreated HHTg rats (Table 1 and Table S1). Compared to Wistar controls, untreated HHTg rats exhibited significantly higher concentrations of pro-inflammatory TNFα, MCP-1, IL-6 and leptin, along with decreased HMW adiponectin levels (Table 2 and Table S1).

**Figure 1:**
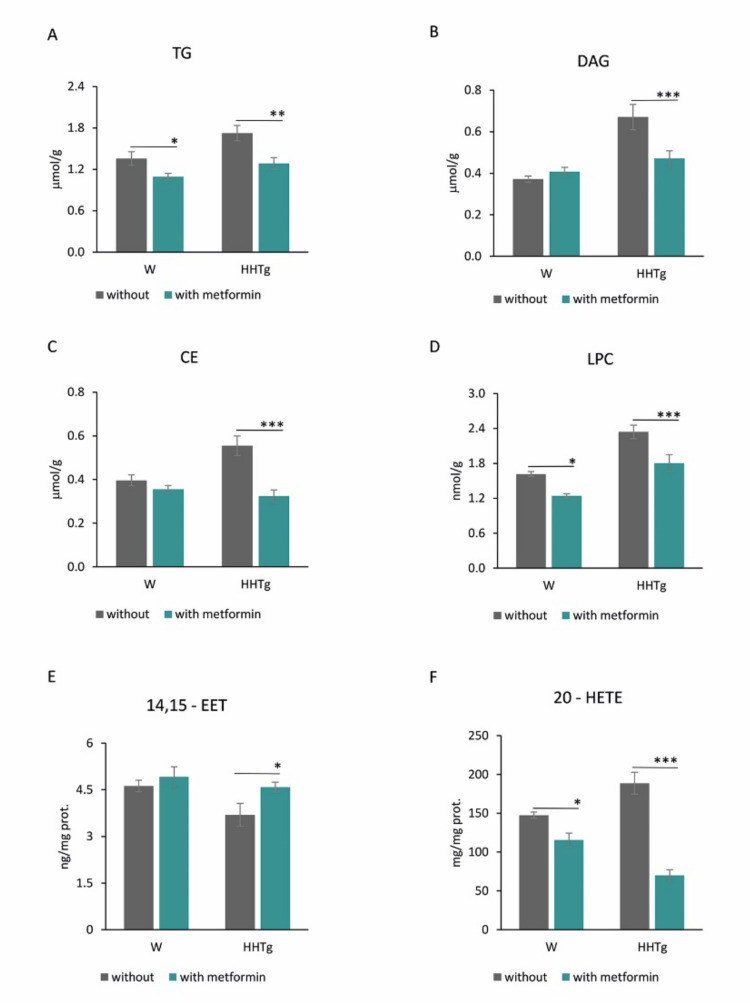
Effects of metformin on myocardial lipids, lipotoxic intermediates (**A**) triglycerides, (**B**) diacylglycerols, (**C**) cholesteryl esters, (**D**) lysophosphatidylcholine) and CYP-derived arachidonic acid metabolites (**E**) 14,15-epoxyeicosatrienoic acid, (**F**) 20-hydroxyeicosatetraenoic acid) in Wistar control (W) and hereditary hypertriglyceridemic (HHTg) rats. Data are mean ± SEM; *n* = 8 for each group. Significance was determined using two-way ANOVA and Fisher’s LSD post-hoc test (* denotes *p* < 0.05; ** denotes *p* < 0.01; *** denotes *p* < 0.001).

**Figure 2:**
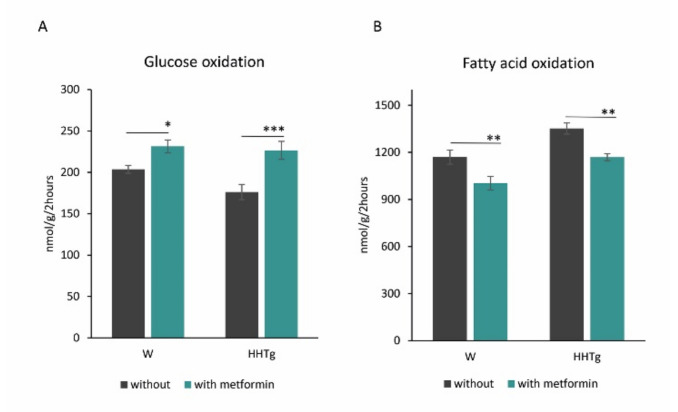
Effects of metformin on glucose (**A**) and palmitate oxidation (**B**) in the myocardium in Wistar control (W) and hereditary hypertriglyceridemic (HHTg) rats. Data are mean ± SEM; *n* = 8 for each group. Significance was determined using two-way ANOVA and Fisher’s LSD post-hoc test (* denotes *p* < 0.05; ** denotes *p* < 0.01; *** denotes *p* < 0.001).

Slightly increased ectopic accumulation of TGs and cholesteryl esters (CE) in the myocardium of HHTg rats was associated with markedly increased levels of myocardial lipotoxic intermediates, such as diacylglycerols (DAG) and lysophosphatidylcholine (LPC) (Figure 1 and Table S2). Untreated HHTg rats exhibited slight alterations in lipid metabolites, namely, decreased 14,15-epoxyeicosatrienoic acid (14,15-EET) and increased 20-hydroxyeicosatetraenoic acid (20-HETE), compared to the control group. Cardiac lipid changes in HHTg rats were associated with increased myocardial FA oxidation, while glucose oxidation tended to decrease (Figure 2 and Table S2).

**Figure 3:**
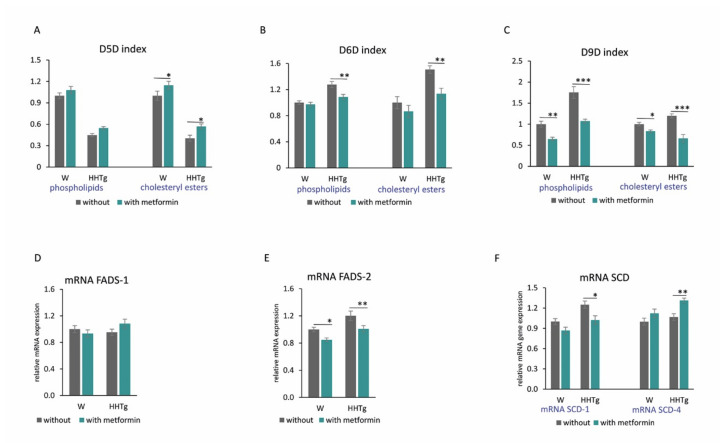
Effects of metformin on relative mRNA expression (**D**–**F**) and activity (desaturation index) (**A**–**C**) of the desaturases SCD-1, FADS-1 and FADS-2 in myocardium of Wistar control (W) and hereditary hypertriglyceridemic (HHTg) rats. Data are mean ± SEM; *n* = 8 for each group. Significance was determined using two-way ANOVA and Fisher’s LSD post-hoc test (* denotes *p* < 0.05; ** denotes *p* < 0.01; *** denotes *p* < 0.001).

**Figure 4:**
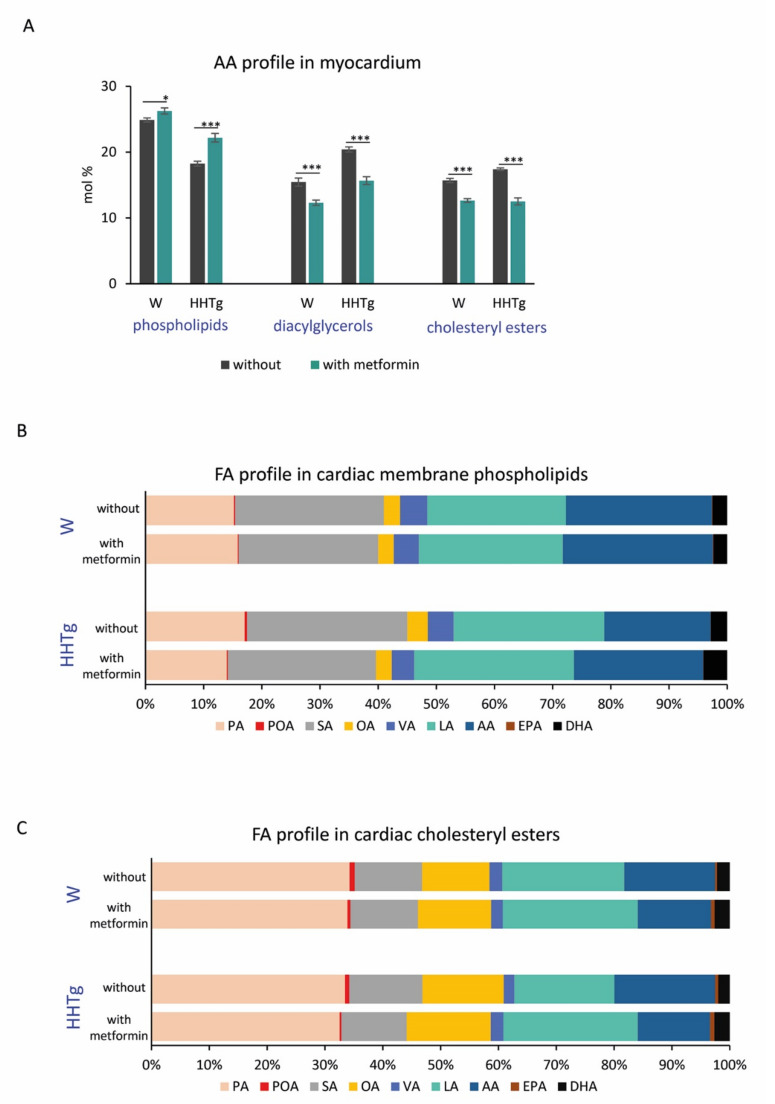
Effects of metformin on arachidonic acid profile in myocardial phospholipids, diacylglycerols and cholestryl esters (**A**), fatty acid profile in myocardial phospholipids (**B**) and cholesteryl esters (**C**) in Wistar control (W) and hereditary hypertriglyceridemic (HHTg) rats; *n* = 8 for each group. Significance was determined using two-way ANOVA and Fisher’s LSD post-hoc test (* denotes *p* < 0.05; *** denotes *p* < 0.001). PA—palmitic acid, POA—palmitoleic acid, SA—stearic acid, OA—oleic acid, VA—vaccenic acid, LA—linoleic acid, AA—arachidonic acid, EPA—eicosapentaenoic acid, DHA—docosahexaenoic acid.

Hypertriglyceridemia in HHTg rats was associated with desaturase system changes in the myocardium. Compared to the Wistar controls, HHTg rats exhibited increased activity indexes for D9-desaturase (D9D) in myocardial phospholipids (PL) as well as cholesteryl esters (CE). In HHTg rats, we observed a significant increase in SCD-1 mRNA gene expression in the myocardium, but SCD-4 mRNA gene expression was unaffected compared to controls (Figure 3 and Table S2). In hypertriglyceridemic rats, D5-desaturase (D5D) activity indexes for both myocardial PLs and CEs markedly reduced. Conversely, D6-desaturase (D6D) activity indexes as well as fatty acid desaturase 2 (FADS-2) mRNA gene expression significantly increased, with fatty acid desaturase 1 (FADS-1) mRNA gene expression remaining unchanged compared to controls (Figure 3 and Table S2). Hypertriglyceridemia was associated with significant changes in PL and CE profiles of FAs in the myocardium (Figure 4). FA composition in myocardial PLs exhibited significantly increased profiles of palmitic (PA, 16:00), palmitoleic (POA, 16:1n7) and linoleic (LA, 18:2n6) acids accompanied by markedly reduced profiles of arachidonic (AA, 20:4n6) and eicosapentaenoic (EPA, 20:5n3) acids (Table S3). FA composition of myocardial CEs in HHTg rats exhibited markedly decreased LA profiles accompanied by significantly increased AA profiles compared to Wistar rats (Table S4).

**Figure 5:**
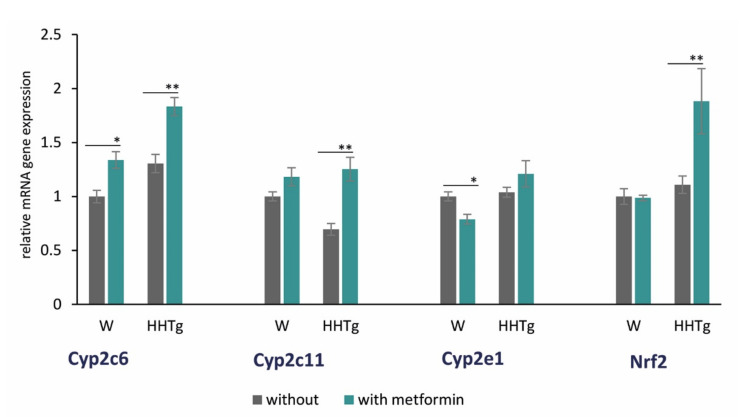
Effects of metformin on relative mRNA expression of some CYP450 family proteins expressed in myocardium and nuclear factor Nrf2 in Wistar control (W) and hereditary hypertriglyceridemic (HHTg) rats. Data are mean ± SEM; *n* = 8 for each group. Significance was determined using two-way ANOVA and Fisher’s LSD post-hoc test (* denotes *p* < 0.05; ** denotes *p* < 0.01).

Hypertriglyceridemia was also associated with alterations in some cardiac-specific CYP isozymes. Increased *Cyp2c6* mRNA gene expression and decreased *Cyp2c11* mRNA gene expression were observed in HHTg rats (Figure 5, Table S2).

### 2.2. Effects of Metformin on Basal Metabolic Parameters and Inflammatory Markers

As shown in Table 1, metformin treatment significantly decreased serum TG concentrations and NEFA levels in HHTg rats, but had no effect on other circulating lipids. As expected, metformin treatment improved markers of glucose tolerance, with AUC_0-180min_ and HOMA-IR significantly decreasing in both rat strains. HHTg rats exhibited decreased non-fasting glucose and EAT weights after metformin administration.

As shown in Table 2, decreased pro-inflammatory TNFα, MCP-1 and IL-6 levels were observed in metformin-treated HHTg rats, with concentrations of leptin or hsCRP remaining unchanged in both strains. Compared to untreated groups, metformin administration resulted in significantly increased serum levels of HMW adiponectin.

### 2.3. Effects of Metformin on Myocardial Lipid Storage and Substrate Utilization

Metformin administration affected substrate utilization in the myocardium. Controls and HHTg rats exhibited significantly reduced myocardial palmitate oxidation, while glucose oxidation in the myocardium increased slightly after metformin administration (Figure 1). Both metformin-treated groups exhibited markedly reduced myocardial TG accumulation (Figure 2). While metformin administration did not affect DAG or CE levels in the Wistar group (despite decreased CE accumulation), we observed markedly reduced levels of toxic lipid intermediates (DAG and LPC) in the myocardium of HHTg rats.

Metformin-treated groups in both strains exhibited significantly reduced levels of pro-inflammatory AA-derived α-hydroxy metabolites (20-HETE). We did, however, observe slightly increased anti-inflammatory 14,15-EET in the HHTg strain compared to untreated rats (Figure 2).

### 2.4. Effects of Metformin on Fatty Acid Desaturases and Profiles in the Myocardium

Metformin treatment significantly altered the activity indexes and gene expressions of desaturase enzymes and markedly changed FA composition in both PL and CE myocardial lipid classes.

The activity indexes for D9D reduced significantly after metformin administration in PL and CE lipid classes in both treated groups (Figure 3). We observed decreased index activity for D6D in metformin-treated HHTg rats in both PL and CE. On the other hand, the activity index for D5D in the myocardium was elevated in metformin-treated controls and HHTg rats based on CE FA composition.

As shown in Figure 3, metformin treatment influenced relative mRNA expression of *SCD-1*, *SCD-4* and *FADS-2* genes (which affect cardiac lipid metabolism) in both strains. In Wistar and HHTg-treated rats, *FADS-2* mRNA expression reduced significantly compared to untreated controls; however, *FADS-1* mRNA expression was not affected (Figure 3). Compared to untreated controls, *SCD-1* mRNA expression in HHTg-treated rats decreased, while *SCD-4* increased. In the metformin-treated Wistar strain, the effect was not significant.

With regard to alterations in FA composition, metformin-treated HHTg rats exhibited decreased saturated fatty acid (SFA) and monounsaturated fatty acid (MUFA) profiles in myocardial PL accompanied by elevated n3 and n6-PUFA profiles. For individual phospholipid FA profiles, we observed significant decreases in the profiles of PA, stearic acid (SA, 18:00), POA, oleic acid (OA, 18:1n9) and vaccenic acid (VA, 18:1n7) in both untreated and metformin-treated HHTg groups. LA, AA, EPA and docosahexaenoic acid (DHA, 22:6n3) profiles were all markedly elevated. LA and EPA profiles increased significantly in treated Wistar rats compared to the Wistar controls (Figure 4).

In myocardial CE, both treated Wistar and HHTg groups exhibited increased LA profiles, in particular accompanied by opposite changes in POA and AA profiles compared to untreated groups (Figure 4). However, we found no significant alterations in the profiles of individual lipid classes (SFA, MUFA, n3 and n6-PUFA) after metformin treatment.

### 2.5. Effects of Metformin on AA Metabolism and Metabolites in the Myocardium

As shown in Figure 5, metformin treatment also affected some cytochrome P450 family members. Relative mRNA expression of *Cyp2c* (*Cyp2c6* and *Cyp2c11*), which converts AA to EET metabolites, increased in HHTg rats after metformin administration. *Cyp2c6* mRNA expression increased in treated Wistar rats, but *Cyp2c11* mRNA expression was not significantly affected (Figure 5). On the other hand, *Cyp2e1* mRNA expression, which is involved in hydroxylase-mediated AA metabolism, reduced significantly in Wistar rats after metformin treatment, with a non-significant alteration observed in the HHTg strain. In addition, mRNA gene expression of transcription factor *Nrf2* (nuclear factor erythroid 2-related factor 2), which also contributes to lipid metabolism, increased markedly in metformin-treated HHTg rats compared to untreated controls (Figure 5). In contrast, the Wistar strain exhibited no significant changes in *Nrf2* mRNA expression.

## 3. Discussion

Myocardial lipid metabolism plays an important role in maintaining proper heart function. Although the heart uses multiple substrates as its source of energy, most of its necessary myocardial energy (70–90%) comes from FA oxidation. With the exception of myocardial TG, cardiac energy metabolism depends on the availability, concentration and composition of circulating NEFA. As previously described [20], the hypertriglyceridemic rat strain exhibits chronically elevated circulating NEFA levels, accompanied by qualitative alterations in NEFA lipid classes, manifesting in both increased SFA and decreased n3-PUFA profiles. Aggravated NEFA metabolism and genetically determined hypertriglyceridemia in HHTg rats combine to promote ectopic lipid deposition in different tissues, including the liver, muscle and heart. This can in turn directly impact cell function, leading to the generation of toxic lipid metabolites such as DAG, ceramides and lysophospholipids [21]. These metabolites interfere with intracellular signaling pathways, resulting in the impairment of metabolic cell functions [22]. Recently, Leonardo P. de Carvalho et al. confirmed in their study the association between plasma ceramide levels and recurrent cardiovascular events or cardiovascular death after acute myocardial infarction (AMI) and supported the idea of a prognostic role of plasma ceramide species after AMI [23]. In our study, the myocardium of HHTg rats exhibited marked accumulation of lipotoxic DAG, which can promote IR and inflammatory pathways in the heart [24].

The desaturase system plays an important role in the regulation of myocardial lipid metabolism [25]. Cardiac functions are also affected by FA composition in myocardial PLs and CEs. In the present study, our prediabetic rat model exhibited alterations in FA profiles (mainly in PLs), as well as in desaturase expression and activity (mainly SCD), factors that can contribute to the impairment of myocardial function in HHTg rats. The FA compositional changes we observed were more pronounced in myocardial PLs than in myocardial CEs, manifesting in increased profiles of POA, OA and LA and decreased profiles of AA and EPA. The EPA:AA ratio is an important cardiovascular marker [26]. Some clinical studies have demonstrated an association between increasing plasma EPA:AA ratios and improved cardiac function, primarily the regulation of inflammatory processes [27].

According to a recent study [28,29], elevated SCD is a strong independent cardiovascular risk factor. In our study, the myocardium of prediabetic hypertriglyceridemic rats exhibited markedly increased SCD-1 gene expression and slightly increased FADS-2 gene expression accompanied by significantly increased D9D and D6D activity indexes, respectively; D5D activity index decreased.

Metformin is a leading treatment for type 2 diabetes, reducing cardiovascular risk independent of its glycemic effect [30]. The mechanisms behind these non-glycemic benefits are not completely clear, but they are understood to be associated with metabolic improvements in lipids and fatty acids [31,32]. In the present study, metformin treatment in our prediabetic rat model had positive effects on myocardial desaturases and fatty acid composition in myocardial phospholipids. Although metformin is considered a possible inhibitor of SCD, few studies have investigated this effect. Zhu et al. [33] found that metformin attenuates triglyceride accumulation in hepatocytes by decreasing SCD-1 expression. However, the cardiac effects of metformin on SCD in the heart have yet to be examined. According to our results, metformin cardiac treatment in HHTg rats markedly reduced SCD-1 activity (based on the D9D desaturation indexes for both phospholipids and the cholesteryl ester lipids class) and gene expression. Markedly reduced SCD-1 in the myocardium after metformin administration was also followed by reduced D6D activity and elevated D5D activity.

On the other hand, gene expression of SCD-4, one of the SCD isoforms exclusively expressed in the heart, increased after metformin administration. We assume this SCD-4 isoform is likely to have a compensatory effect due to the lack of SCD-1 in the heart. Results from studies on leptin-deficient *ob*/*ob* mice [34] suggest that the SCD-4 isoform is specifically regulated by leptin. Therefore, the metabolic effect of leptin in the myocardium may be mediated by SCD expression, pointing to the specific role of SCD-4 in cardiac physiology. Leptin represses the gene expression of SCD-4 but not SCD-1. However, since the SCD-4 gene is not sensitive to PUFA repression [34], it is unlikely that SCD-4 has a significant effect on the regulation of myocardial lipid metabolism.

On the other hand, SCD-1 is an important factor in the maintenance of proper cardiac lipid metabolism through the regulation of lipogenesis and lipolysis in cardiomyocytes. SCD-1 is also understood to reprogram myocardial metabolism and improve cardiac function [6] by shifting substrate utilization in the heart. In our study, reduced myocardial SCD after metformin treatment was associated with increased glucose oxidation and decreased FA oxidation in the heart. These results correspond with those of another study, which found that a lack of SCD-1 expression increases glucose transport and oxidation while decreasing FA uptake and oxidation in the heart [35]. Although in our study, the expressions of genes involved in glucose and FA oxidation were not assessed, a study by Dobrzyn et al. [35] found that disruption of the SCD1 gene in leptin-deficient *ob/ob* mice was associated with the reduced expression of genes involved in FA transport and lipid synthesis in the heart. However, in vitro studies of isolated cardiomyocytes have documented the opposite effects of metformin on substrate utilization, notably increased FA oxidation and glycolysis [15]. Based on the above results, we assume that metformin has the potential to positively affect cardiac metabolic flexibility via SCD, enabling substrates to switch and thus maintain better fuel efficiency. It has been shown that cardiomyocytes can flexibly use different substrates depending on their availability or energy requirements [36]. Metabolic adaptability in the myocardium is impaired in IR and associated with an imbalance between lipid and glucose uptake by cardiomyocytes, leading to intramyocardial lipid accumulation and lipotoxicity [22].

According to one study, SCD-1 knockout significantly affects myocardial substrate utilization and protects the heart from cardiac ectopic lipid deposition in SCD-1 knockout mice [37]. Another study found that ectopic lipid deposition in the myocardium may lead to functional impairments, as observed in obese ZDF *db*/*db* mice and leptin-deficient *ob/ob* mice [38].

In our study, metformin treatment improved lipid accumulation and metabolism in the myocardium of prediabetic rats. It is acknowledged that the beneficial effects of metformin are mediated by the activation of adenosine AMP-kinase [15]. The activation of transcriptional factor Nrf2 can be another mechanism that contributes to the beneficial effect of metformin on myocardial lipid metabolism and that can ameliorate cardiovascular risk [39]. In addition to improving oxidative stress, Nrf2 can also affect and regulate lipid metabolism by inhibiting lipogenesis and influencing the gene expression of acetyl-CoA carboxylase (ACC), fatty acid synthase (FAS) and 3-hydroxy-3-methylglutaryl-coenzyme A reductase (HMGCR). In our study, increased cardiac gene expression of Nrf2 may have been involved in the alteration of myocardial FA profiles observed after metformin administration. Through these changes in fatty acid profiles in the myocardium, metformin can also affect FA-induced signaling pathways of insulin sensitivity and inflammation [40]. In our study, neither the protein level of Nrf2 nor the downstream regulatory genes were investigated, which can be considered as a limitation of the study.

Interestingly, the majority of fatty acids that undergo β-oxidation in the heart are not SFA but MUFA. The preferred MUFA substrate is oleic acid (OA). In the heart, oleic acid plays an important role in the regulation of fatty acid oxidation, but its mechanisms are poorly understood. Oleate, the conjugate base of oleic acid, activates oxidative pathways [41]. In agreement, in our study, we found an association between reduced OA profiles in myocardial phospholipids after metformin treatment and decreased fatty acid oxidation in the heart. According to one study of transgenic mice with an overexpression of cardiac peroxisome proliferator-activated receptor α (PPARα), one of the possible mechanisms linking oleate with fatty acid oxidation is the activation of PPARα [42]. However, increased phosphorylation and activity of AMPK in the heart may also be involved. As oleate is easily incorporated into phospholipids, increased OA profiles after metformin may be responsible for FA oxidation in the heart.

**Figure 6:**
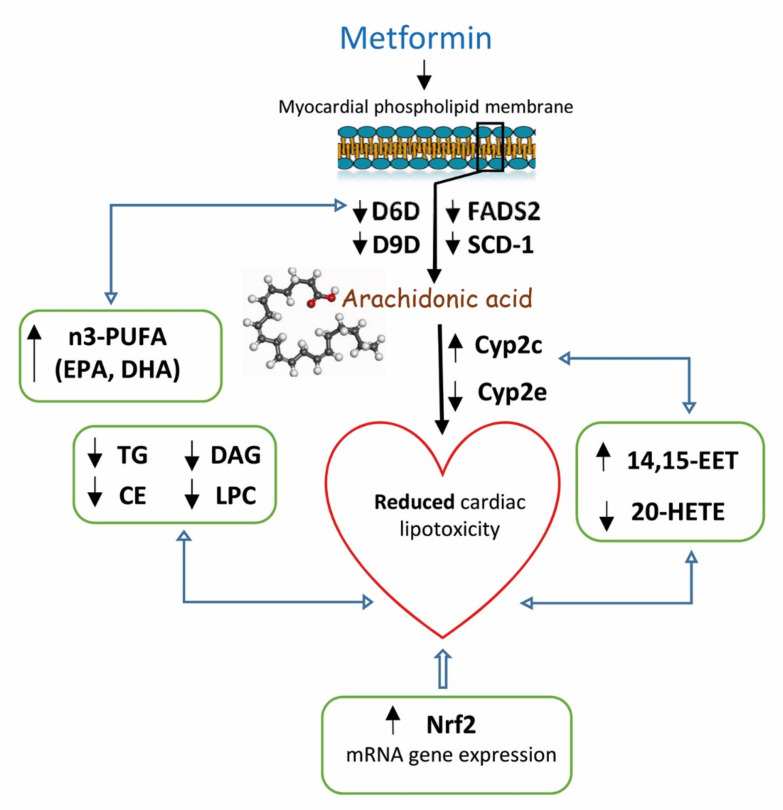
Putative involvement of metformin in cardiac arachidonic metabolism, CYP-derived arachidonic acid metabolites and some cytochrome P450 proteins. The alterations in AA metabolism is manifested in the form of elevated production of anti-inflammatory 14,15-EET, produced by Cyp2c and inhibited formation of pro-inflammatory 20-HETE, produced by Cyp2e. In addition, reduced SCD-1 expression with an inhibited D9D activity index in the myocardium contributes to reduced myocardial accumulation of neutral lipids and toxic lipid intermediates, respectively. Affecting the desaturation system is associated with an altered fatty acid profile in membrane phospholipids in the myocardium, which contributes to an increased proportion of n3-PUFA, resulting in increased membrane fluidity. All these changes, together with markedly elevated *Nrf2* expression, contribute to the reduction of cardiac lipotoxicity and inflammation.

Based on our results (as shown in Figure 6), reduced AA profiles in myocardial phospholipids in prediabetic rats after metformin treatment resulted in markedly increased FA profiles. However, metformin had an opposite effect on AA profiles in the other myocardial lipid class: DAGs and CEs. AA was metabolized by various metabolic pathways into bioactive lipid mediators. Depending on the production type, quantity and timing, these mediators can have numerous physiological and pathophysiological effects on the heart. It is therefore possible that the differences in AA profiles we observed were related to the capacity of different metabolic pathways to produce lipid mediators with beneficial as well as detrimental cardiovascular properties. The CYP enzyme system is understood to be one of the possible AA metabolic pathways, but little is known about its activity in the myocardium [43]. In our study, metformin treatment increased the gene expression of *CYP2c*, one of the most predominant cardiac CYP epoxygenases. CYP-derived AA metabolites play an important role in maintaining proper cardiac function. While EETs possess anti-inflammatory, thrombolytic and angiogenic properties, other 20-HETE metabolites have pro-inflammatory effects on cardiac function [7]. In this study, we found that metformin had positive effects on CYP-derived AA metabolites in the myocardium, increasing 14,15-EET and markedly decreasing 20-HETE. By influencing the gene expression of *CYP2c* and altering AA profiles of individual lipid classes in the heart, metformin affects the production of EET and HETE, leading to improved cardiac function. No study has investigated the effect of metformin on EET and HETE concentrations. However, it is possible that nuclear factor-κB (NF-κB) and mitogen-activated protein kinase (MAPK) contribute to the effects of metformin on CYP-derived AA metabolites [44].

In addition, EPA and DHA compete with AA in the processes of membrane PL incorporation [45] and CYP enzyme conversion [7]. EPA can act as an inhibitor by competing for cyclooxygenase (COX) and lipoxygenase (LOX) enzymes, resulting in the formation of less potent inflammatory metabolites, such as those formed from AA [46]. AA and its metabolites also enter numerous metabolic pathways that connect lipid metabolism with inflammation and, under certain conditions, may even assist in alleviating inflammation.

## 4. Materials and Methods

### 4.1. Animals and Diet

All experiments were performed in agreement with the Animal Protection Law of the Czech Republic (311/1997), which is in compliance with European Community Council recommendations (86/609/ECC) on the use of laboratory animals, and approved by the Ethics Committee of the Institute for Clinical and Experimental Medicine (Protocol Number: 64873/2016-3/OVZ-30.0, date: 03/11/2016). The study was performed using 6-month-old male Wistar rats (obtained from Charles River, Kohl, Germany) as the control group and 6-month-old male hereditary hypertriglyceridemic (HHTg) rats (provided by the Institute for Clinical and Experimental Medicine, Prague, Czech Republic) as the non-obese prediabetic model. Rats were kept in temperature- (22 °C) and humidity-controlled conditions under a 12-h/12-h light/dark cycle with free access to food (maintenance diet for rats and mice; Altromin, Lage, Germany) and drinking water. Wistar and HHTg rats were randomized into groups with or without metformin treatment mixed as part of a standard diet at a dose of 300 mg/kg b.wt. for 4 weeks.

At the end of the experiment, rats were sacrificed by decapitation after light anesthesia (zoletil 5 mg/kg b.wt.) in a postprandial state. Aliquots of serum and tissue samples were collected and stored at −80 °C for further analysis.

### 4.2. Analytical Methods and Biochemical Analysis

Serum levels of triglycerides, glucose and NEFA, as well as total and HDL cholesterol were measured using commercially available kits (Erba Lachema, Brno, Czech Republic and Roche Diagnostics, Mannheim, Germany).

Serum insulin, glucagon, leptin and HMW adiponectin concentrations were determined using rat ELISA kits (Mercodia AB, Uppsala, Sweden; BioVendor, Brno, CZ; MyBioSource, San Diego, CA, USA). Circulating MCP-1 and TNFα, as well as IL-6 and hsCRP concentrations were also measured using rat ELISA kits (Bio-Source International, San Diego, CA, USA; eBioscience, Vienna, Austria; Alpha Diagnostics International, San Antonio, Tx, USA, respectively).

For the oral glucose tolerance test (OGTT), blood glucose was determined after a glucose load (300 mg/100g b.wt.) administered intragastrically after overnight fasting. Blood was drawn from the tail before the glucose load at 0 min and 30, 60, 120 and 180 min thereafter.

To determine triglycerides, diacylglycerols, cholesteryl esters and lysophosphatidylcholine in the myocardium (left ventricle), samples were extracted in dichloromethane/methanol. The resulting pellet was dissolved in isopropyl alcohol and isolated by thin-layer chromatography. The content of individual lipid classes was determined by enzymatic assay (Erba-Lachema, Brno, Czech Republic; Roche Diagnostics, Germany). Concentrations of 14,15-EET and 20-HETE in the myocardium were determined using rat ELISA kits (MyBioSource, USA).

Glucose and palmitate oxidation in the myocardium were measured ex vivo in the left ventricle of the myocardium based on incorporation of the radiolabeled substrates ^14^C-U glucose and ^14^C-palmitate into CO_2_, as described previously [47].

### 4.3. Fatty Acid Composition and Fatty Acid Desaturases Activity

Fatty acid levels were reported as a percentage of the total fatty acids. For determination of fatty acid composition in the heart (left ventricle), samples were extracted in dichloromethane/methanol followed by the addition of KH_2_PO_4_; the solution was then centrifuged. The organic phase was evaporated under N_2_ and the resulting pellet dissolved in an isopropyl alcohol/hexane mixture. Individual lipid classes were separated by thin-layer chromatography using hexane-diethyl ether-acetic acid (70:30:1, *v*/*v*) as a solvent system, then extracted from silica gel, and finally converted to fatty acid methyl esters (FAME) using 1% sodium methoxide in dry methanol. FA profiles in separated lipid classes were established by gas chromatography using the Hewlett-Packard GC system with hydrogen as the carrying gas, a flame ionization detector and a carbowax-fused silica capillary column (Varian, Palo Alto, CA, USA). Individual FAME peaks were identified by comparing retention times with those of authentic standards (mix of fatty acids, Restek, PA, USA) [48].

Fatty acid desaturase activity was calculated based on fatty acid composition in individual lipid classes. The following product/precursor ratios were used to reflect the enzyme activities involved in fatty acid metabolism: D5-desaturase (20:4n6/20:3n6), D6-desaturase (18:3n6/18:2n6) and D9-desaturase (16:1n7/16:0) [49,50].

### 4.4. Relative mRNA Expression

Total RNA was isolated from the heart’s left ventricle using RNA Blue (Top-Bio, Vestec, Czech Republic). The concentration and purity of RNA was assessed by NanoDrop spectrophotometer (NanoDrop^TM^ 2000, ThermoFisher Scientific, Waltham, MA, USA). Reverse transcription and quantitative real-time PCR analysis was performed using the TaqMan RNA-to-CT 1-Step Kit and TaqMan Gene Expression Assay (Applied Biosystems, Waltham, MA, USA) and carried out using a ViiATM 7 Real-Time PCR System (Applied Biosystems, USA). Relative expressions were determined after normalization against Hprt as the internal reference and calculated using the 2-ΔΔCt method. Results were run in triplicate.

### 4.5. Statistical Analysis

Two-way ANOVA was used to analyze the individual and combined effects of treatment and strain for treatment-*versus*-strain interactions. All data were analyzed of normal distribution. Fisher’s LSD post-hoc test was used for variables showing evidence of treatment-*versus*-strain interactions. The test was adjusted for multiple comparisons to determine whether metformin treatment would significantly influence parameters of cardiac lipid metabolism and lipid intermediates in HHTg and Wistar strains. The student’s *t*-test was used to determine the effect of hypertriglyceridemia on metabolic parameters and lipid metabolism in the heart before metformin treatment. Statistical significance was determined at a value of *p* < 0.05. All results are expressed as mean ± SEM. Statistical analysis was performed using StatSoft Statistica 14 (StatSoft CZ; Prague, Czech Republic).

## 5. Conclusions

Our results demonstrate that metformin treatment markedly affects fatty acid composition in myocardial phospholipids, as well as myocardial desaturase gene expression and activity in a prediabetic rat model. In addition, metformin decreases the accumulation of neutral lipids, as well as the lipotoxic intermediates diacylglycerols and lysophosphatidylcholine. The inhibition of myocardial SCD-1 and FADS-2 with decreased d9D and d6D activity may also lead to a reduced accumulation of lipids and lipotoxic intermediates in the myocardium.

Moreover, metformin can affect arachidonic acid metabolism and CYP-derived AA metabolites. Increased cardiac gene expression of *CYP2c*, a predominant cytochrome P450 isoform in the myocardium, was associated with increased 14,15-EET and markedly reduced 20-HETE. All these effects may contribute to the beneficial cardioprotective and anti-inflammatory effects of metformin.

## Figures and Tables

**Table 1:** Basal metabolic and morphological parameters in Wistar control (**W_WITHOUT_**) and hereditary hypertriglyceridemic (**HHTg_WITHOUT_**) rats after metformin administration (**W_WITH METFORMIN_**), (**HHTg_WITH METFORMIN_**).

	W_WITHOUT_	W_WITH METFORMIN_	HHTg_WITHOUT_	HHTg_WITH METFORMIN_	P_STRAIN_	P_TREATMENT_	P_INTERACTION_
Body weight (g)	428 ± 12	432 ± 10	411 ± 9	395 ± 6	<0.01	n.s.	n.s.
LV weight (g/100 g b.w.)	0.131 ± 0.006	0.135 ± 0.005	0.134 ± 0.003	0.143 ± 0.004	n.s.	n.s.	n.s.
EAT weight (g/100 g b.w.)	1.235 ± 0.034	1.196 ± 0.061	1.839 ± 0.038	1.536 ± 0.048 ***	<0.001	<0.001	<0.01
Non-fasting glucose (mmol/L)	6.6 ± 0.2	6.6 ± 0.1	8.6 ± 0.2	8.1 ± 0.2 *	<0.001	n.s.	n.s.
AUC_0-180min_ (mmol/L)	1268 ± 22	1176 ± 13 **	1476 ± 18	1417 ± 13 *	<0.001	<0.001	n.s.
HOMA-IR	2.1 ± 0.1	1.8 ± 0.1 *	2.7 ± 0.1	2.3 ± 0.1 **	<0.001	<0.001	n.s.
Insulin (nmol/L)	0.279 ± 0.022	0.240 ± 0.036	0.273 ± 0.028	0.238 ± 0.041	n.s.	n.s.	n.s.
Glucagon (pg/mL)	224.61 ± 14.82	212.51 ± 12.33	191.22 ± 13.38	158.84 ± 4.94	<0.01	n.s.	n.s.
Serum TG (mmol/L)	1.56 ± 0.11	1.03 ± 0.08 *	3.45 ± 0.26	2.65 ± 0.20 **	<0.001	<0.001	n.s.
Serum cholesterol (mmol/L)	1.51 ± 0.07	1.37 ± 0.06	1.77 ± 0.03	1.84 ± 0.03	<0.001	n.s.	<0.05
HDL-C (mmol/L)	1.27 ± 0.08	1.15 ± 0.06	0.88 ± 0.03	0.91 ± 0.03	<0.001	n.s.	n.s.
NEFA (mmol/L)	0.496 ± 0.020	0.449 ± 0.022	0.713 ± 0.015	0.646 ± 0.017 *	<0.001	<0.01	n.s.

Two-way ANOVA results: P_STRAIN_ denotes the significance of W vs. HHTg (strain effects); P_TREATMENT_ denotes the significance of metformin treatment (treatment effects); P_INTERACTION_ denotes the significance of metformin treatment in both strains (treatment vs. strain interaction). For multiple comparisons (metformin treatment vs. non-treated controls), Fisher’s LSD post-hoc test was used; * denotes *p* < 0.05; ** denotes *p* < 0.01; *** denotes *p* < 0.001. Data are mean ± SEM; *n* = 8 for each group. LV—left ventricle; EAT—epididymal adipose tissue.

**Table 2:** Inflammatory markers and adipocytokines in Wistar control (**W_WITHOUT_**) and hereditary hypertriglyceridemic (**HHTg_WITHOUT_**) rats after metformin administration (**W_WITH METFORMIN_**), (**HHTg_WITH METFORMIN_**).

	W_WITHOUT_	W_WITH METFORMIN_	HHTg_WITHOUT_	HHTg_WITH METFORMIN_	P_STRAIN_	P_TREATMENT_	P_INTERACTION_
TNFα (pg/mL)	2.280 ± 0.259	2.738 ± 0.435	11.488 ± 0.697	8.709 ± 0.280 ***	<0.001	<0.05	<0.01
MCP-1 (ng/mL)	4.794 ± 0.242	4.873 ± 0.236	10.552 ± 1.492	6.744 ± 0.525 **	<0.001	<0.05	<0.05
hsCRP (mg/mL)	1.253 ± 0.069	1.457 ± 0.086	1.304 ± 0.049	1.329 ± 0.058	n.s.	n.s.	n.s.
IL-6 (pg/mL)	126.5 ± 12.5	120.9 ± 8.9	171.0 ± 8.3	144.0 ± 3.6 *	<0.001	n.s.	n.s.
Leptin (ng/mL)	5.51 ± 0.64	5.45 ± 0.55	7.51 ± 0.22	6.59 ± 0.16	<0.01	n.s.	n.s.
HMW Adiponectin (μg/mL)	1.015 ± 0.034	1.358 ± 0.068 ***	0.893 ± 0.030	1.245 ± 0.072 ***	<0.05	<0.001	n.s.

Two-way ANOVA results: P_STRAIN_ denotes the significance of W vs. HHTg (strain effects); P_TREATMENT_ denotes the significance of metformin treatment (treatment effects); P_INTERACTION_ denotes the significance of metformin treatment in both strains (treatment vs. strain interaction). For multiple comparisons (metformin treatment vs. non-treated controls), Fisher’s LSD post-hoc test was used; * denotes *p* < 0.05; ** denotes *p* < 0.01; *** denotes *p* < 0.001. Data are mean ± SEM; *n* = 8 for each group.

## Data Availability

All datasets generated for this study are included in the article/Supplementary Materials.

## Supplementary Materials

The following materials (Tables S1–S4) can be found at https://www.mdpi.com/article/10.3390/ijms22147680/s1.

## Abbreviations

AA	arachidonic acid
ACC	acetyl-coenzyme A carboxylase
AMPK	adenosine monophosphate-activated protein kinase
CE	cholesteryl ester
COX	cyclooxygenase
CYP450	cytochrome P450
DAG	diacylglycerol
D5D	delta 5-desaturase
D6D	delta 6-desaturase
D9D	delta 9-desaturase
EET	epoxyeicosatrienoic acid
FA	fatty acid
FADS	fatty acid desaturase
FAS	fatty acid synthase
HETE	hydroxyeicosatetraenoic acid
HMGCR	3-hydroxy-3-methylglutaryl-coenzyme A reductase
hsCRP	high-sensitivity C-reactive protein
IL-6	interleukin 6
LOX	lipoxygenase
LPC	lysophosphatidylcholine
MAPK	mitogen-activated protein kinase
MCP-1	monocyte chemoattractant protein-1
NEFA	non-esterified fatty acid
NF-κB	nuclear factor-κB
NRF2	nuclear factor erythroid-2-related factor 2
PL	phospholipid
PPARα	peroxisome proliferator-acivated receptor α
SCD	stearoyl-coenzyme A desaturase
SREBP1	sterol regulatory element-binding protein 1
TG	triglyceride
TNFα	tumor necrosis factor α
