# Supplementary material for: Metformin Affects Cardiac Arachidonic Acid Metabolism and Cardiac Lipid Metabolite Storage in a Prediabetic Rat Model
Source: Int J Mol Sci. 2021 Jul 19;22(14):7680. doi: 10.3390/ijms22147680 (PMC8305829; doi:10.3390/ijms22147680)
Supplement: Supplementary file 1 [file ijms-22-07680-s001.zip › ijms-1267151-supplementary.pdf]

SUPPLEMENTARY TABLE 1 – Serum parameters

	W	HHTg	P
Body weight (g)	428.13 ± 11.51	410.63 ± 9.17	n.s.
ETT weight (g/100g b.w.)	1.235 ± 0.034	1.839 ± 0.038	<0.001
Non-fasting glucose (mmol/l)	6.563 ± 0.191	8.638 ± 0.181	<0.001
AUC_{0-180} (mmol/l)	1268 ± 22	1476 ± 18	<0.001
HOMA-IR	2.095 ± 0.083	2.667 ± 0.110	<0.01
Insulin (nmol/l)	0.279 ± 0.022	0.273 ± 0.028	n.s.
Glucagon (pg/ml)	224.61 ± 14.82	191.22 ± 13.38	n.s.
Serum TG (mmol/l)	1.559 ± 0.108	3.446 ± 0.256	<0.001
Serum cholesterol (mmol/l)	1.506 ± 0.067	1.773 ± 0.034	<0.01
HDL-C (mmol/l)	1.265 ± 0.082	0.883 ± 0.030	<0.01
NEFA (mmol/l)	0.496 ± 0.020	0.713 ± 0.015	<0.001
TNFα (pg/ml)	4.289 ± 0.301	13.337 ± 0.921	<0.001
MCP-1 (ng/ml)	4.794 ± 0.242	10.552 ± 1.492	<0.01
IL-6 (pg/ml)	126.501 ± 12.453	171.047 ± 8.329	<0.05
Leptin (ng/ml)	5.513 ± 0.635	7.510 ± 0.217	<0.05
HMW Adiponectin (μg/ml)	1.015 ± 0.034	0.893 ± 0.030	<0.05

SUPPLEMENTARY TABLE 2 – Parameters of lipid metabolism in the myocardium

	W	HHTg	P
TG in heart (μmol/g)	1.357 ± 0.099	1.726 ± 0.112	<0.05
DAG in heart (μmol/g)	0.372 ± 0.015	0.671 ± 0.061	<0.01
CE in heart (μmol/g)	0.397 ± 0.025	0.555 ± 0.045	<0.05
Lysophosphatidylcholine (μmol/g)	1.617 ± 0.041	2.341 ± 0.115	<0.001
14,15 – EET (ng/mg prot.)	4.623 ± 0.185	3.696 ± 0.366	<0.05
20 – HETE (ng/mg prot.)	147.433 ± 4.075	188.709 ± 14.092	<0.05
Glucose oxidation (nmol/g/g/2h)	203.59 ± 4.73	176.19 ± 9.15	<0.05
Fatty acid oxidation (nmol/g/g/2h)	1169.72 ± 45.56	1351.73 ± 35.73	<0.01
FADS1 ($2^{-\Delta\Delta Ct}$)	1.000 ± 0.053	0.954 ± 0.047	n.s.
FADS2 ($2^{-\Delta\Delta Ct}$)	1.000 ± 0.033	1.202 ± 0.071	<0.05
SCD1 ($2^{-\Delta\Delta Ct}$)	1.000 ± 0.045	1.250 ± 0.053	<0.01
SCD4 ($2^{-\Delta\Delta Ct}$)	1.000 ± 0.051	1.068 ± 0.049	n.s.
CYP2c6 ($2^{-\Delta\Delta Ct}$)	1.000 ± 0.050	1.305 ± 0.067	<0.05
CYP2c11 ($2^{-\Delta\Delta Ct}$)	1.000 ± 0.037	0.696 ± 0.043	<0.01
D5D index (PL)	1.000 ± 0.114	0.447 ± 0.021	<0.001
D6D index (PL)	1.000 ± 0.078	1.276 ± 0.047	<0.001
D9D index (PL)	1.000 ± 0.204	1.754 ± 0.138	<0.001
D5D index (CE)	1.000 ± 0.181	0.405 ± 0.043	<0.001
D6D index (CE)	1.000 ± 0.261	1.507 ± 0.058	<0.001
D9D index (CE)	1.000 ± 0.124	1.198 ± 0.049	<0.01

SUPPLEMENTARY TABLE 3 – Fatty acid profile in cardiac phospholipids

	W	HHTg	P
Palmitic acid (mol %)	15.088 ± 0.231	17.050 ± 0.287	<0.001
Palmitoleic acid (mol %)	0.209 ± 0.018	0.420 ± 0.036	<0.001
Stearic acid (mol %)	25.321 ± 0.826	27.545 ± 0.233	<0.05
Oleic acid (mol %)	2.794 ± 0.149	3.524 ± 0.153	<0.01
Vaccenic acid (mol %)	4.633 ± 0.214	4.436 ± 0.116	n.s.
Linoleic acid (mol %)	23.628 ± 0.394	25.915 ± 0.358	<0.001
Arachidonic acid (mol %)	24.882 ± 0.307	18.250 ± 0.372	<0.001
Eicosapentaenoic acid (mol %)	0.064 ± 0.003	0.025 ± 0.001	<0.001
Docosahexaenoic acid (mol %)	2.534 ± 0.165	2.835 ± 0.062	n.s.

SUPPLEMENTARY TABLE 4 – Fatty acid profile in cardiac cholesteryl esters

	W	HHTg	P
Palmitic acid (mol %)	34.264 ± 0.366	33.476 ± 0.718	n.s.
Palmitoleic acid (mol %)	0.891 ± 0.116	0.722 ± 0.046	n.s.
Stearic acid (mol %)	11.625 ± 0.529	12.653 ± 0.143	n.s.
Oleic acid (mol %)	11.681 ± 0.921	14.068 ± 0.552	<0.05
Vaccenic acid (mol %)	2.188 ± 0.161	1.839 ± 0.142	n.s.
Linoleic acid (mol %)	21.110 ± 0.551	17.302 ± 0.583	<0.001
Arachidonic acid (mol %)	15.689 ± 0.326	17.395 ± 0.204	<0.001
Eicosapentaenoic acid (mol %)	0.359 ± 0.063	0.559 ± 0.066	<0.05
Docosahexaenoic acid (mol %)	2.194 ± 0.127	1.986 ± 0.156	n.s.
